# Stepped-wedge cluster randomised trial of a smoking cessation counselling training programme for midwives treating women with functional health illiteracy and low socioeconomic status (PROMISE): a study protocol

**DOI:** 10.1186/s13063-020-04555-0

**Published:** 2020-07-07

**Authors:** Jeroen Bommelé, Linda Springvloet, Naïma Abouri, Karianne Djoyoadhiningrat-Hol, Margriet van Laar, Matthijs Blankers

**Affiliations:** 1 Trimbos Institute, The Netherlands Expertise Centre for Tobacco Control, Utrecht, the Netherlands; 2 Pharos, Utrecht, the Netherlands; 3 Lung Foundation Netherlands, Amersfoort, the Netherlands

**Keywords:** Smoking, Pregnancy, Midwifery, Protocol, PROMISE, Randomised controlled trial

## Abstract

**Background:**

In the Netherlands, midwives are required to use the ‘V-MIS’ (Minimal Intervention Strategy for Midwives) smoking cessation counselling protocol to help pregnant women quit smoking. This counselling protocol is often poorly implemented in midwifery practices. It may also be less suitable for pregnant woman with low socioeconomic status or functional health illiteracy. We created an adapted version of the V-MIS protocol that is intended to facilitate implementation in midwifery practices: PROMISE (PROtocol for growing up smokefree using a Minimal smoking cessation Intervention Strategy in the Early stages of life). For this adapted protocol, midwives use carbon monoxide meters, storyboard leaflets, and specific communication techniques for women with functional health illiteracy. They will receive a face-to-face training in using these materials and communication techniques.

**Methods:**

The effectiveness and implementation of PROMISE will be tested in a stepped-wedge cluster randomised controlled trial. We will randomise clusters of midwifery practices and departments in hospitals. We will then train them, subsequently, at regular intervals (‘steps’). At each step, practices that will receive training cross over from the control condition to the experimental condition. We will measure how well the PROMISE protocol has been implemented by assessing the rate of pregnant women that received detailed smoking cessation counselling from their midwives (primary outcome). Our secondary target group is pregnant women with functional health illiteracy and low socioeconomic status. Among them, we will assess smoking status and health-related outcome before and after pregnancy.

**Discussion:**

The PROMISE smoking cessation counselling protocol is intended to help midwives, OB-GYNs, and other obstetrics professionals to support pregnant women with smoking cessation.

**Trial registration:**

Dutch Trial Registry: NTR 6305/NL6158. Registered on 20 December 2016.

## Background

Smoking tobacco during and after pregnancy is a major preventable cause of perinatal deaths and serious health problems, such as miscarriage, foetal growth problems, low birth weight, and congenital heart defects [1–4]. In addition, children of smoking parents are at higher risk of developing nicotine dependence later in life and are more likely to be exposed to second-hand and third-hand smoke [5, 6]. This puts them at higher risk for health problems during infancy and makes them more likely to contract bronchial infections, meningitis, and asthma [7–11]. In the Netherlands, about 75 children start smoking every day [12].

While many (future) parents quit smoking before pregnancy or in the early stage of pregnancy, smoking among (future) parents is still prevalent in many countries [11]. In the Netherlands, 7.4% of pregnant women smoke during pregnancy [13]. Smoking during pregnancy is particularly common among women with a low socioeconomic status (SES) [14–18]. In the Netherlands, 16% of low SES pregnant women smoke during pregnancy, while 12% of middle-educated and 3% of highly educated women do [13]. Moreover, relapse after delivery is high: about 50 to 70% of women who quit smoking before or during pregnancy relapse within 6 to 12 months after giving birth, and about one third of women who quit in an early stage of their pregnancy will relapse during the second or third trimester [11, 18–20].

Primary obstetric care professionals play an important role in smoking cessation care for pregnant women in the Netherlands. The vast majority of pregnant women regularly visit either a midwife (primary care) or an OB-GYN (secondary care), and most receive post-natal care and ‘youth health care’ after giving birth [21]. Youth health care is a set of free municipal care services for children aged 0–18 [22].

Midwives have a 4-year professional degree, which includes over 18 months of clinical work [23]. They are trained in educating prospective parents about pregnancies and accompanied women in their pregnancy, birth, and post-natal period. They work autonomously in uncomplicated pregnancies. Only when health problems arise or are likely to arise will they refer clients to OB-GYNs in hospitals. OB-GYNs are medical doctors specialised in both gynaecology and obstetrics. Both midwives and OB-GYNs are completely covered under the national medical insurance system. Because almost all pregnant women visit either a midwife or an OB-GYN, the Dutch evidence-based guideline for treating pregnant women with tobacco addiction stresses the importance of obstetric care professionals in providing smoking cessation advice to pregnant smoking women and in preventing relapse after delivery [24].

Dutch midwives are required to use the ‘V-MIS’ smoking cessation protocol (Minimal Intervention Strategy for Midwives) [25–27]. Using this protocol is optional for OB-GYNs. The V-MIS is an effective, minimal intervention protocol for guiding pregnant women and their partners in smoking cessation. Based on a randomised controlled trial (RCT) in 1996, 12% of smoking pregnant women counselled with V-MIS remained continuously abstinent during pregnancy, versus 3% in the usual care group [28]. Despite its efficacy, the V-MIS protocol has not been well implemented in daily practice [29, 30]. Midwives tend to go through only a few of the seven steps of the protocol. This is likely due to a lack of time and communication skills [29].

OB-GYNs, post-natal care, and youth health care are also required to implement protocols for smoking cessation support, but they may opt for protocols other than the V-MIS protocol [25]. In the Netherlands, post-natal care nurses are trained at a vocational level and support mothers at home during the first 8 days after delivery (3–10 h per day). They educate mothers about breastfeeding, do regular health checks on both mother and child, and do various domestic chores for them. This post-natal care is completely covered under national medical insurance, and most Dutch women receive post-natal care after childbirth [21]. Dutch youth health care is a municipal health care system in the Netherlands that provides free national and preventive care services to young people aged 0–18. Parents of young children (0–12 years) visit the youth health care clinic regularly, where nurses and paediatricians check their children’s social, physical, and cognitive development [22]. Youth health care nurses educate parents about parenting and vaccinate young children against common diseases.

Both primary and secondary obstetric care in the Netherlands are coordinated through regional obstetric partnerships (in Dutch: ‘verloskundig samenwerkingsverbanden’). These partnerships usually consist of a single large hospital and the midwifery practices in its surrounding region. In many cases, post-natal care and youth health care are included in the partnership as well. Local obstetric partnerships provide a relevant platform for disseminating new smoking cessation protocols among obstetric professionals.

The Dutch Health Care Inspectorate recently concluded that multidisciplinary smoking cessation care has been poorly implemented in both primary and secondary obstetric care, post-natal care nurses, and youth health care [22, 27, 29]. This has been illustrated by the fact that in 2016, 27% of youth health care professionals did not discuss smoking cessation with smoking parents [22, 31]. Besides being poorly implemented, the current smoking cessation protocols also appear less suitable for people with low SES or functional health illiteracy. While smoking during pregnancy is most prevalent among low SES women [18, 32], it is important that smoking cessation counselling is sensitive to this target client group. This is even more the case for those with functional health illiteracy. It is estimated that about 12% of Dutch adults have poor functional health literacy skills and experience difficulties in interpreting health-related information and in communicating with health professionals [33]. It is therefore imperative that smoking cessation protocols are adapted for women with low SES or poor functional health literacy skills.

The ‘PROtocol for growing up smokefree using a Minimal smoking cessation Intervention Strategy in the Early stages of life’ (PROMISE) aims to address some of the limitations of the V-MIS smoking cessation protocol and focusses on effective communication towards pregnant women with low SES or poor functional health literacy skills. In order to assess whether PROMISE manages to address these limitations and prompts midwives and OB-GYNs towards effectively discussing smoking cessation with smoking pregnant women, we will perform a stepped-wedge RCT. This paper describes the development, content, and procedures of the PROMISE protocol and the design of the stepped-wedge RCT.

## Methods/design

### Study summary

For this study, we will train midwives and OB-GYNs to use the PROMISE protocol for helping pregnant women to quit smoking. These care professionals will use carbon monoxide meters, storyboard leaflets, and specific communication skills for women with functional health illiteracy. Our main objective is to improve the rate at which they discuss smoking cessation strategies with pregnant women. Using a stepped-wedge randomised controlled design, we will evaluate the effectiveness of the PROMISE protocol while it is being implemented in 23 midwifery practices and 3 obstetrics hospital departments across three regions in the Netherlands. Over the course of the study period, we will assess the rate at which midwives and OB-GYN discuss smoking cessations strategies with their clients (primary outcome). We will also collect data about the level of appreciation of PROMISE by all professionals (i.e. midwives, OB-GYNs, post-natal care nurses, and youth health care; secondary outcome). Finally, we will assess smoking status and other perinatal parameters among pregnant clients before and after delivery.

### Aims and hypothesis

The main goal of this study is to examine the effectiveness of the PROMISE protocol in obstetric care. We test whether PROMISE will improve the level of smoking cessation support by obstetric care professionals and whether these professionals become more effective in delivering this support. Our primary outcome measure is the rate at which primary obstetric care professionals (midwives and OB-GYNs) discuss specific smoking cessation strategies with pregnant women.

*Hypothesis*: We hypothesise that the rate at which midwives and OB-GYNs discuss specific smoking cessation strategies will increase after professionals have been trained to use the PROMISE protocol.

### Study design

**Fig. 1:**
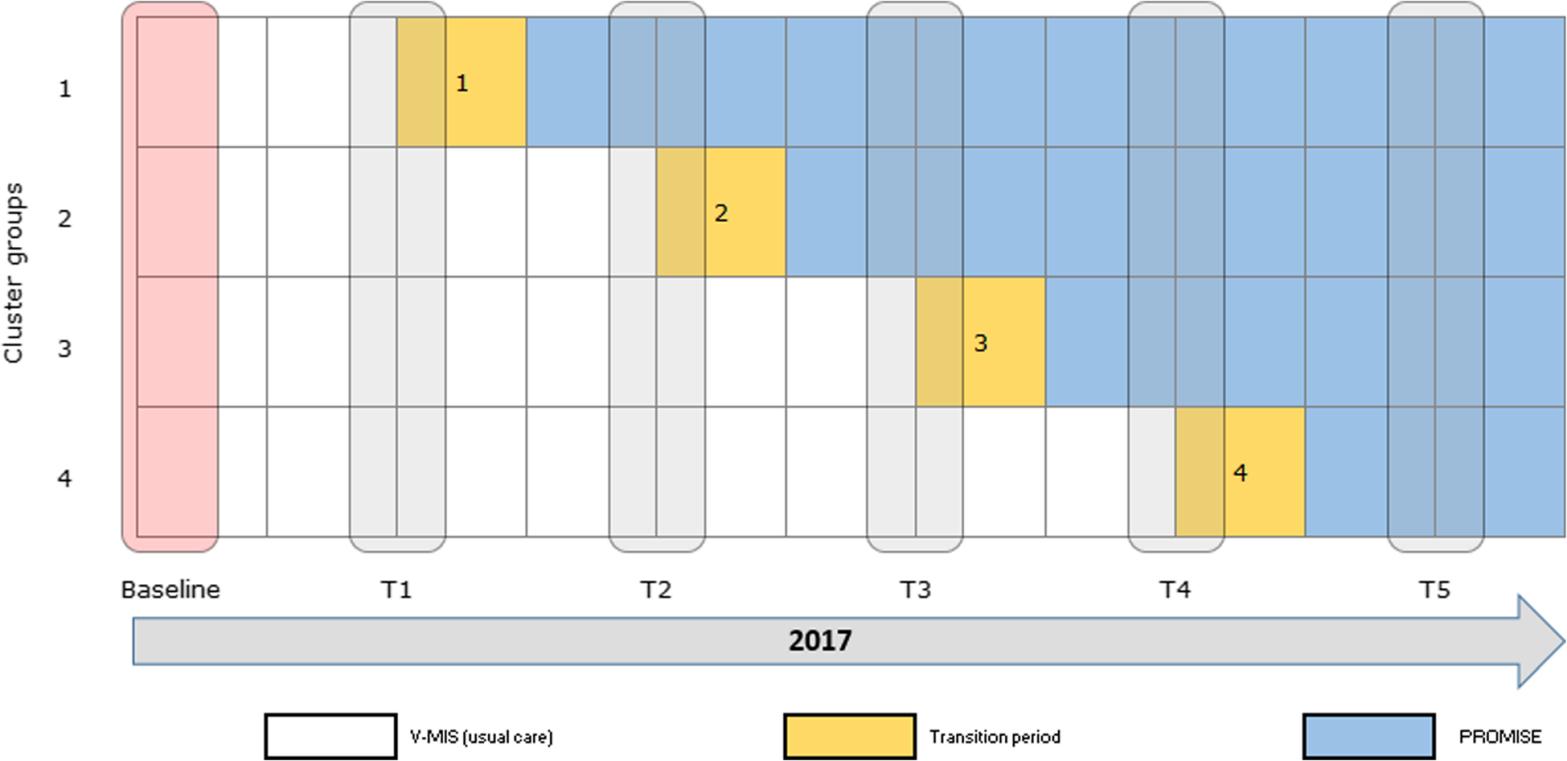
Overview of clusters of midwifery practices. Midwifery practices from three regions will be randomly allocated to one of four steps. At each step, practices that received training cross over from the control condition (V-MIS/usual care) to the experimental condition (PROMISE)

We will use a stepped-wedge randomised controlled design to implement the PROMISE protocol in 23 midwifery practices and 3 obstetrics hospital departments across three regions in the Netherlands: Amsterdam-Noord, Zaanstreek-Waterland, and Heerenveen. In a stepped-wedge cluster RCT, a programme is gradually implemented among clusters of respondents (see Fig. 1) [34]. In this study, clusters of professionals in midwifery practices or hospitals will continue to use the current V-MIS protocol at first (usual care, control condition) and will switch to using the PROMISE protocol after receiving an extensive face-to-face training (experimental condition).

We will train clusters of midwives and OB-GYNs, subsequently, at four regular intervals (‘steps’). At each step, practices that received training cross over from the control condition to the experimental condition. We will measure outcomes at baseline, at each of the four steps (2 months apart, step 1 starting 2 months after baseline) and 2 months after the last step.

This study has been registered in the Dutch Trial Registry (NTR 6305) and was exempt from medical ethics review by the Ethics Committee of the Erasmus Medical Centre in Rotterdam (MEC-2016-605).

### Study procedure

#### Regions

We have selected three regions for implementing the PROMISE protocol. Eligible regions (1) had at least 250 smoking pregnant women with low SES in the previous year; (2) were willing and capable to implement PROMISE within its obstetric partnership; (3) had a well-organised level of cooperation between midwives, OB-GYNs, post-natal care nurses, and youth health care; and (4) were not participating in any other tobacco-related research projects.

#### Midwives and OB-GYNs

After receiving informed consent from midwives and OB-GYNs, we will randomly assign their practices to one of four steps. We will follow a training and cross-over procedure in accordance to our stepped-wedge study design. At baseline, all clusters of practices will use the existing V-MIS protocol for supporting women to quit smoking (i.e. control group). Two months after baseline, we will train the first cluster of practices (i.e. step 1) and will provide them with the PROMISE materials. From that moment onwards, this first cluster will cross over to the experimental condition and will continue to use the new PROMISE protocol. We will train clusters 2, 3, and 4 subsequently, each at a 2-month interval. After training, these clusters also cross over to the experimental condition. This way, we will train all practices in 4 steps over a 10-month period and all practices will be using the PROMISE protocol from month 8 onwards. By gradually implementing the PROMISE protocol among clusters of practices, we will replicate a real-life implementation of protocols that require face-to-face training. The order at which practices will receive the face-to-face training will be randomised at the practice level and stratified by region. We had no inclusion or exclusion criteria for the midwives and OB-GYNs, other than being employed at one of the practices enrolled in our study. At the start of the study (January 2017), participating midwives will complete a baseline questionnaire (T0) about their level of support to smoking pregnant women. They will receive a similar questionnaire at each step of the study (T1–T4) and will receive a final questionnaire 2 months after the final step (T5). These questionnaires include our main outcome variables. Figure 1 provides a schematic overview of the training steps. Care professionals will receive no incentive for filling out the questionnaires, but those who complete the training will receive continuing education credits.

#### Post-natal care nurses and youth health care

Post-natal care nurses and youth health care professionals will be offered a face-to face training too. Contrary to the midwife training, their training will only focus on preventing relapse after pregnancy. As the performance of post-natal care nurses and youth health care professionals will not affect the main outcome of this study, the order at which post-natal care nurses and youth health care professionals receive training will not be randomised stratified by region. Instead, their training will be provided at a first come, first served basis. Post-natal care nurses and youth health care professionals will receive a baseline questionnaire before the training and one follow-up questionnaire 12 months after training. These questionnaires will be used for evaluating the materials and improving future materials for these professionals. Participants will receive no incentive for filling out the questionnaire, but those who complete the training will receive continuing education credits.

#### Pregnant women

Pregnant women will be recruited through the midwifery practices and hospitals. Eligible women (1) are 18 years or older, (2) have been 0–3 months pregnant, (3) are current smokers or have quit smoking within the 6 months before pregnancy, and (4) have been registered in a practice or hospital that takes part in PROMISE. We will have no exclusion criteria. Midwives and OB-GYNs will provide recruitment materials to potential participants and inform them about the study. After providing written informed consent through their midwife of OB-GYNs, participants will receive a baseline questionnaire after 8 weeks of pregnancy and follow-up questionnaires at 2 weeks after delivery (T1) and at 6 months after delivery (T2). In those questionnaires, we assess smoking status and ask about the support they received from their midwife or OB-GYNs, post-natal care nurse professional, and youth health care professional. Women will receive a gift voucher of 10 euros for each questionnaire they complete.

This study was conducted from January 2017 to December 2019. All professionals have participated from January 2017 onwards, and the first training session has begun in March 2017. Pregnant women were able to enrol until March 2018. Among care professionals, we collected data from January 2017 to December 2017 and among pregnant women from January 2017 to December 2019.

### Randomisation

We will use R 3.0 to randomise the order (step) at which midwifery practices and hospitals will be trained. Within each of the three regions, practices will be randomly allocated to one of the four training steps. Neither professionals nor pregnant women will be blinded to their allocation. The authors will also not be blinded to the allocated arm that participants are in.

### Sample size

We used the package ‘SWSamp’ in R 3.0 to calculate the power and minimal sample size for the four-cluster stepped-wedge RCT [35, 36]. Our main outcome is the percentage of pregnant smoking women with whom midwives or OB-GYNs discussed specific smoking cessation strategies. We calculated the sample size needed for a power of .80. Assuming a differential effect 35% (control/V-MIS condition) vs. 60% (PROMISE condition) coverage of addressing smoking during the health consultations [29, 30], and taking into account *α* = 0.05 (2-sided) and a coefficient of variation (CV) between clusters of 0.3, we need 18 professionals in each cluster (region in our case). Since there are three regions, we will need at least 54 midwives/OB-GYNs to complete our questionnaires. We will enrol as many professionals as possible.

### Control condition: V-MIS protocol

**Fig. 2:**
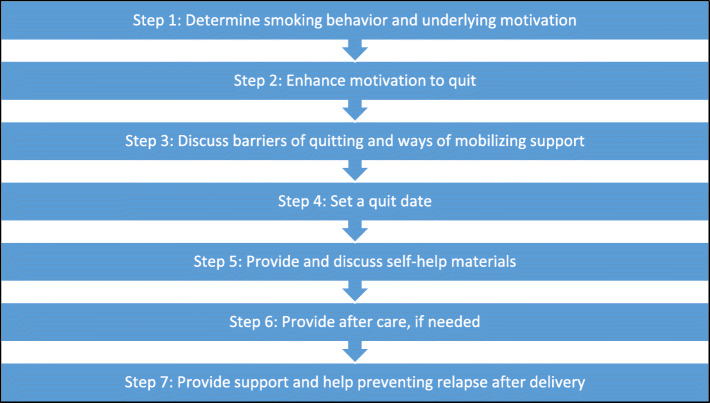
Counselling steps of the V-MIS protocol for smoking cessation care among pregnant women

All participating midwifery practices and hospitals start in the control condition and will use the V-MIS protocol (usual care), for which they have already been trained in the past. The V-MIS protocol consists of seven consecutive counselling steps in which midwives and their clients discuss smoking cessation. Depending on their client’s motivation to quit smoking, midwives could take multiple steps in a single visit. While these counselling steps could best be conducted in consecutive order, deviations are allowed (e.g. going back a step). Figure 2 provides an overview of the counselling steps of the V-MIS protocol. A more extensive description of the V-MIS protocol can be found elsewhere [28].

#### Step 1: Determine smoking behaviour and underlying motivations

Midwives assess their client’s smoking status, number of cigarettes per day, and whether the client is motivated to quit within 30 days. Smoking status is registered in the online system.

#### Step 2: Increase motivation to quit

If a client is not motivated to quit smoking, midwives use motivational interviewing techniques to strengthen their client’s intrinsic motivation to quit smoking. They also discuss short-term advantages of quitting and disadvantages of smoking.

#### Step 3: Discuss barriers of quitting and ways of mobilising support

If a client is motivated to quit, midwives discuss barriers to successful quitting. Together, the client and midwife discuss how to mobilise social support for quitting.

#### Step 4: Set a quit date

Together, the midwife and client set a quit date. Midwives register the quit date in the client’s records and discuss the quit attempt during the next session.

#### Step 5: Provide and discuss self-help materials

While setting a quit date, the midwife and client discuss whether the client needs additional support. Midwives may provide self-help materials or refer the client to a specific type of behavioural support (i.e. telephone counselling, personal coaching, or group counselling). The use of nicotine replacement therapy is not encouraged in the Netherlands among pregnant women and may only be used among specific groups of heavy smokers [24].

#### Step 6: Provide after care, if needed

If a client has quit smoking, midwives continue to discuss smoking (cessation) in the following visits in order to prevent relapse. They discuss past moments of craving and ways to deal with them. If a client has relapsed, midwives try to motivate their client to quit smoking again.

#### Step 7: Provide support and help preventing relapse after delivery

Midwives could help prevent relapse after delivery by discussing this beforehand with their clients. In the last stages of pregnancy, the client and the professional discuss which barriers and difficult situations the client may encounter. The midwives stimulate their client to develop strategies for overcoming these barriers. They also discuss the dangers of second-hand smoke to the baby.

### Experimental protocol: PROMISE

Those switched to the experimental condition will start using the PROMISE protocol. The PROMISE protocol consists of both the original V-MIS protocol (including its seven steps) and additional elements designed to save consultation time and to increase counselling effectiveness. The content of the seven steps has been made more suitable to pregnant women with low SES or functional health illiteracy.

#### Carbon monoxide meters

We added the use of carbon monoxide (CO) measurements to steps 1 and 6 of the V-MIS protocol. A carbon monoxide meter measures the CO level in someone’s expired air and can be used to determine smoking status during pregnancy [37]. While CO levels continue to be elevated for at least 24 h after smoking a cigarette, CO meters are able to determine smoking status even if someone abstained from smoking a few hours prior to measurement. CO measures offer a non-invasive way to show pregnant women how much CO potentially reaches her unborn child. CO measurements are mandatory in smoking cessation care for pregnant women in some countries [38], but they are rarely used in Dutch midwifery practices. This study is the first one to explore the use of CO measurements among pregnant women in the Netherlands.

We will train midwives to use the CO measurement as a conversation starter and to avoid using the measurement outcome in a disapproving or criticising way. They will use it to stimulate smoking cessation (step 1) or staying smoke free (step 6). We will provide specific PROMISE score forms illustrating how CO and nicotine from cigarettes reach the unborn baby. Midwives could use this to communicate the health effect of smoking to women with functional health illiteracy. In line with previous research [37], midwives will be trained to use a cut-off point of 4 part per million to identify smokers among their clients.

#### Storyboard leaflets

We developed storyboard leaflets to help midwives enhance motivation among women with functional health illiteracy (steps 2 and 3), to guide these women towards smoking cessation (step 5), and to prevent relapse after quitting (step 6). Storyboard leaflets are visual stories, accompanied by a small text. By using simple texts and illustrations, information can be made accessible and comprehensible to both people with either poor or good literacy skills [39]. Storyboard leaflets stimulate doctor-client communication and have proven useful in other health-related domains, such as diabetes and obesity [40, 41]. They may be used for counselling for both people with poor literacy skills and for people with good literacy skills [40].

**Fig. 3:**
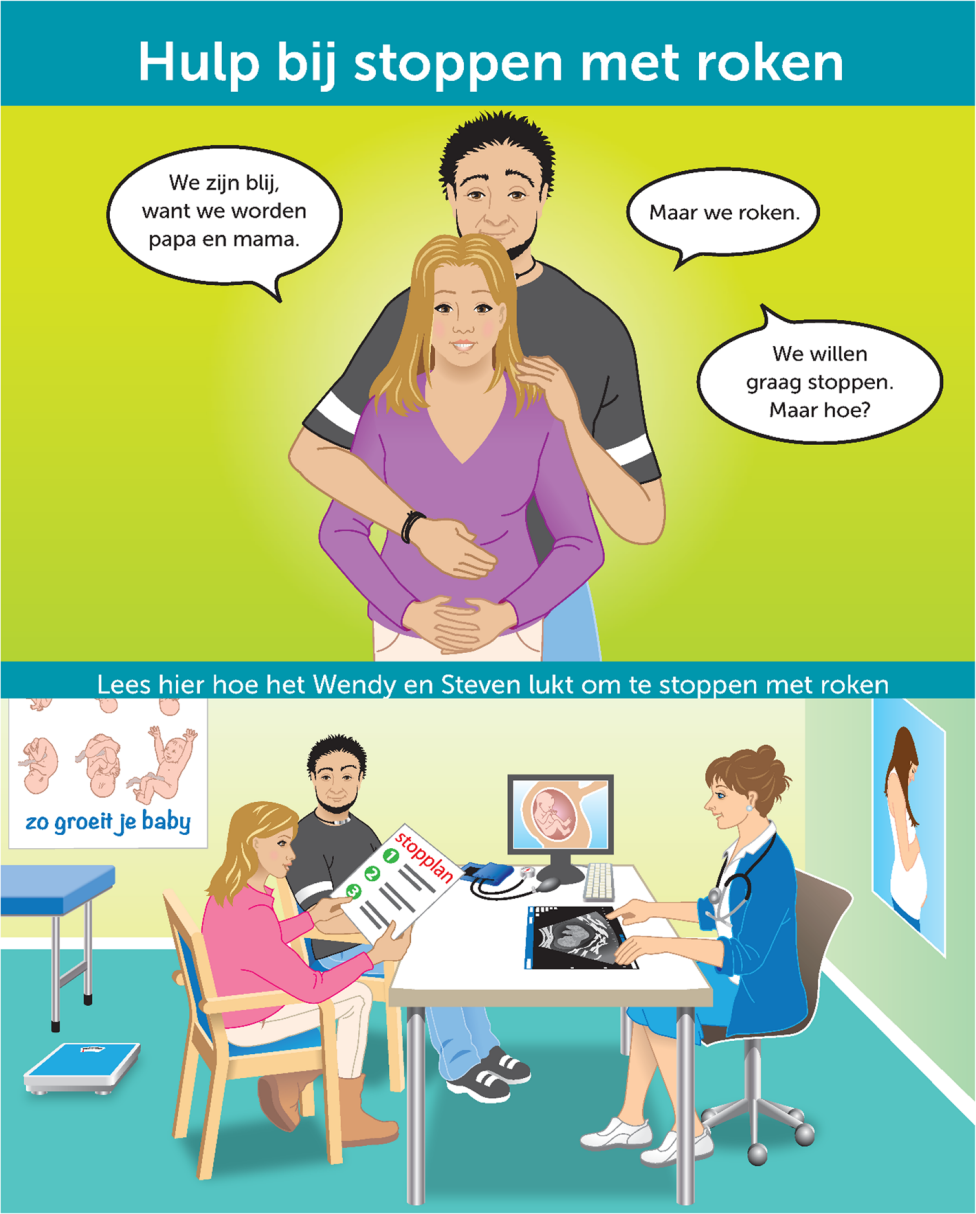
Example pages from a PROMISE storyboard leaflet

In an exploratory study, we interviewed 23 pregnant women (of whom most had a low SES) and asked them to help developing the story line for the leaflets. Their feedback made the texts and illustrations highly suitable for women with poor functional health literacy skills. Based on the target group’s feedback, we developed three versions of the same storyboard: one for pregnant women with a partner, one for pregnant women without a partner, and one for women who recently gave birth (see Fig. 3 for example images). Midwives will use the first two versions; post-natal care nurses and youth health care professionals will use the third one.

#### Referral to smoking cessation support

In the original V-MIS protocol, midwives were only able to refer clients to intensive smoking cessation counselling in step 5. In the PROMISE protocol, they will be able to do so from step 2 onwards. By allowing them to refer women earlier on in the protocol, they are able to help clients even if they lack time to counsel them themselves. As midwives are the main care provider for pregnant women, we will stress that midwives should only refer clients if they see no other option. To stimulate the use of the referrals in such cases, we developed an online list of local referral options for each of the three regions (a social map).

#### Relapse prevention: post-natal care and youth health care

Since most women who quit smoking during pregnancy relapse after delivery [9, 20, 32], relapse prevention is extremely important. Unexpected stress factors may arise after delivery and could increase the chance of relapse [42]. While midwives and OB-GYNs visit their clients 1–3 times after delivery, relapse prevention has been a small part of the original V-MIS protocol. Post-natal care nurses spend more time with clients after delivery (8 days, 3–10 h per day), and mothers visit the youth health care clinic about 15 times in the first 4 years of their children’s life. Both post-natal care nurses and youth health care therefore play an important role in guiding new mothers and preventing relapse after childbirth.

Although both types of professionals do not influence the main study outcome, we felt a strong moral obligation to help these professionals too and to allow continued smoking cessation support after delivery. This is why post-natal care nurses and youth health care professionals will be offered both a face-to-face training and a counselling manual too. Contrary to the training and manual for midwives, their training and manual will be focussed at preventing relapse after delivery (instead of stimulating smoking cessation). We also developed specific storyboard leaflets about remaining smoke free after delivery. Post-natal care nurses and youth health care professionals could use this to support young mothers in remaining abstinent.

To further stimulate cooperation between midwives, OB-GYNs, post-natal care nurses, and youth health care, we developed a new transferring document. Health professionals could use this form to exchange information about the smoking status and smoking cessation.

#### Face-to-face training and counselling manual

To stimulate the correct use of the PROMISE protocol, all midwives, OB-GYNs, post-natal care nurses, and youth health care professionals will be offered a comprehensive face-to-face training. Although some midwives and OB-GYNs might have received a training in the V-MIS protocol years before this study, the materials used in that training were outdated. That is why we developed a new training with newer materials.

The training for midwives and OB-GYNs will consist of four parts. First, they receive background information about smoking cessation and the PROMISE protocol. Next, they will receive background information about people with functional health illiteracy. This section includes ways of recognising whether a client has poor literacy skills and techniques for communicating with them. One such technique is the teach-back method, in which a health professional checks whether a client understands the professional’s recommendations by asking the client to summarise the professional’s recommendations in their own words [43]. Third, we will teach motivational interviewing techniques to strengthen communicating with low SES and functionally illiterate women and their partners [44]. Participants will practice example conversations and discuss past challenging conversations with clients. Finally, participants will be trained in using the carbon monoxide meter.

The training for post-natal care nurses and youth health care professionals will only include the background information and the motivational interviewing techniques. Both post-natal care nurses and youth health care professionals will not use the carbon monoxide meter. By leaving out information about low health literacy, we avoid making the post-natal care nurses uncomfortable, as they often have poor literacy skills themselves.

All training participants will receive a comprehensive counselling manual. This manual discusses the V-MIS counselling protocol and the elements we added. It provides information about people with poor functional health literacy skills and ways of using the carbon monoxide meter. It includes a large number of example conversations about common barriers, difficult situations, withdrawal symptoms, and ways of mobilising social support. All example conversations are based on motivational interviewing techniques. We have developed three manuals: one for midwives and OB-GYNs, one for post-natal care nurses, and one for youth health care.

### Outcomes

Table 1 represents an overview of all outcomes collected among professionals and pregnant women/mothers. All outcomes for pregnant women measure their current status. Health status, for example, is measured by asking ‘How healthy do you feel?’ with response options (1) ‘Healthy’, (2) ‘Sometimes healthy, sometimes unhealthy’, and (3) ‘Unhealthy’.

**Table 1:** Outcome measures

	Measurements^a^		
**Midwives and OB-GYNs**	T0	T1–T4	T5
Background characteristics	x		
Number of smoking clients seen in past month	x	x	x
% smoking cessation discussed	x	x	x
% specific/individual strategies for smoking cessation discussed	x	x	x
Use of protocol components	x	x	x
Barriers and facilitators of using protocol elements	x	x	x
Transferral of smoking status to post-natal care	x	x	x
Appreciation of PROMISE			x
**Post-natal care and youth health care**	T0	T1	
Background characteristics	x		
Use of protocol components^b^	x	x	
Transfer of smoking status from midwife	x	x	
Transfer of smoking status between post-natal care and youth health care	x	x	
Appreciation of PROMISE		x	
**Pregnant women/mothers**	T0	T1	T2
Background characteristics	x		
Smoking status	x	x	x
Partner’s smoking status	x	x	x
Exposure to environmental smoke	x	x	x
Perceived health status	x	x	x
Self-efficacy	x	x	x
Perceived health effects of smoking	x	x	x
Baby background characteristics		x	
Appreciation of protocol elements		x	x
Use of protocol elements by midwife		x	
Use of protocol elements by post-natal care		x	
Use of protocol elements by youth health care			x

^a^Midwives and OB-GYNs: follow-up at each step (every 2 months); post-natal care nurses and youth health care: before training (T0) and 1 year after training (T1); pregnant women/mothers: after 8 weeks of pregnancy (T0), 2 weeks after delivery (T1), and 6 months after delivery

^b^Motivational interviewing techniques only

#### Measurements

**Fig. 4:**
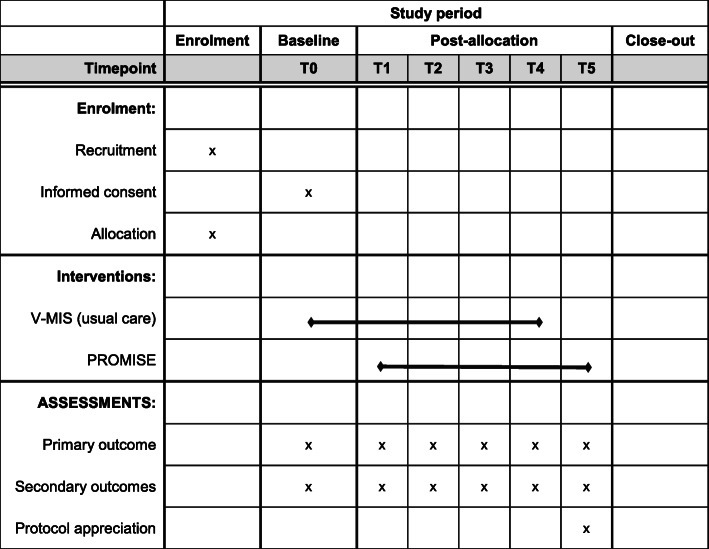
Schematic overview of the data collection for midwives and OB-GYNs. Midwives and OB-GYNs will receive training in four steps. They will receive a follow-up questionnaire at each step

Professionals and pregnant women will be asked to fill out brief questionnaires. Midwives and OB-GYNs will fill out six questionnaires: one at baseline, four after each step (i.e. at each 2-month interval of the stepped-wedge design), and a sixth one 2 months after the last training. Professionals in both post-natal care and youth health care will fill out two questionnaires: one before they are trained with PROMISE and one at the end of 2018 (approximately 12 months after training). Pregnant women will fill out three questionnaires, containing questions about the smoking cessation care they receive during and after pregnancy. These questionnaires could be filled out online, via telephone, or on paper. An overview of the data collection among midwives can be found in Table 1 and Fig. 4.

#### Primary outcome

Our primary outcome measure is change in percentage of smoking pregnant women with whom midwives (or OB-GYNs) discussed specific quit-smoking strategies. We use the self-reported data from the questionnaires filled out by the midwives and OB-GYNs for this. We will calculate this outcome measure by dividing the number of women with whom the professional discussed specific smoking cessation strategies by the total number of smoking women seen by the professional. This will result in a percentage at each time point for each individual professional. We expected that this percentage will increase over time in the total sample, as more professionals will use the PROMISE protocol and will be better able to support smoking clients.

#### Statistical analyses

We will use a Generalised Linear Mixed Modelling analysis in SPSS 25+ or R 3.0+ to analyse sample-wide changes in our primary outcome over time. In stepped-wedge designed studies, data should be interpreted at population level [34]. This means that not individual changes, but sample-wide changes over time are used to test the effectiveness of the new protocol (similar to a repeated cross-sectional design). In our study, all care professionals fill out a questionnaire at each of the six time points. In this questionnaire, they indicate the number of smoking women they have seen in the past month and the number whom they provided detailed smoking cessation counselling.

We calculate the percentage of smoking women who received detailed counselling for each care professional at each of the six time points. As each care professional will attend one of four training sessions, all professionals switch from the usual care condition (control) to the experimental condition at one of those time points.

Using a Generalised Linear Mixed Modelling (GLMM) analysis, we will analyse whether professionals provided detailed counselling to a higher portion of their smoking clients after receiving the training than before. We will include the factors ‘cluster’ (i.e. practices) and ‘individual’ (i.e. midwife/OB-GYN) as random effects, and ‘step’ will be included as fixed (time) effect. The exact link function (e.g. nominal, binominal) for the model will be based on the outcome variable. We will conduct an intention-to-treat analysis to control for lost to follow-up. Only members of the research team will have access to the research data.

As we do not anticipate any adverse effects in any of the two intervention arms, we will not conduct any interim analyses to determine if the trial needs to be stopped.

## Discussion

This article describes the study protocol for testing the effectiveness of the PROMISE smoking cessation counselling protocol for midwives and OB-GYNs. The PROMISE protocol is an adapted version of the existing V-MIS protocol and focusses on effective communication towards pregnant women with low SES or poor functional health literacy skills. Using a stepped-wedge randomised controlled design, we will evaluate the effectiveness of the PROMISE protocol while we implement it in 23 midwifery practices and three obstetrics hospital departments across three regions in the Netherlands. Over the course of the training period, we will assess the rate at which midwives and OB-GYN discuss smoking cessation strategies with smoking clients.

This study will be the first to introduce both carbon monoxide and storyboard leaflets for smoking cessation at a large scale among obstetrics care professionals. Both professionals and their clients will be unfamiliar with these materials. Although implementing the new protocol may be challenging at times, this study will deliver valuable insights into the added value of using these new materials and protocol in the Dutch context.

While midwives and OB-GYNs are the main study population of this project, we will develop extensive materials for post-natal care nurses and youth health care professionals too. By involving all obstetrics care professionals, we will create a systematic and comprehensive protocol for multidisciplinary smoking cessation support for pregnant women and young mothers. By motivating all care professionals to use similar communication techniques, support materials, and transferral documents, we might help reducing exposure to tobacco smoke among new-borns. Unfortunately, we will be unable to properly assess the level cooperation between professionals in this study. Future research might study ways of improving cooperation between all partners in local obstetric partnerships.

The results of this study will be submitted to an international peer-reviewed journal. If effective, the PROMISE protocol can be incorporated in the existing smoking cessation support protocols for pregnant women in the Netherlands. This will in turn motivate obstetrics care professionals throughout the Netherlands to provide smoking cessation support more frequently and help improving the health of mothers and their children.

## Data Availability

Not applicable.

## Abbreviations

CO: Carbon monoxide
OB-GYN: Obstetrics and gynaecology doctor
PROMISE: PROtocol for growing up smokefree using a Minimal smoking cessation Intervention Strategy in the Early stages of life
RCT: Randomised controlled trial
SES: Socioeconomic status
V-MIS: Minimal Intervention Strategy for Midwives (Dutch: Verloskundige Minimale Interventiestrategie)
